# Demographic determinants of self-medication in the population covered by health centers in Tabriz

**DOI:** 10.15171/hpp.2019.26

**Published:** 2019-08-06

**Authors:** Hamid Reza Shaamekhi, Mohammad Asghari Jafarabadi, Mahasti Alizadeh

**Affiliations:** ^1^Department of Community and Family Medicine, Faculty of Medicine, Tabriz University of Medical Sciences, Tabriz, Iran; ^2^Road Traffic Injury Research Center, Tabriz University of Medical Sciences, Tabriz, Iran; ^3^Department of Biostatistics and Epidemiology, Faculty of Health, Tabriz University of Medical Sciences, Tabriz, Iran; ^4^Social Determinants of Health Research Centre, Health Management and Safety Promotion Research Institute, Tabriz University of Medical Sciences, Tabriz, Iran

**Keywords:** Cause, Drug, Demographic, Self-medication

## Abstract

**Background: ** Self-medication is the choice and use of medications by people to treat a self-diagnosed illness or symptom. The aim of this study was to search the relationship between a number of socio-demographic conditions and self-medication in the population covered by health complexes in Tabriz, Iran.

**Methods: ** This study was a cross-sectional descriptive-analytic study and was carried out on the population covered by health complexes in Tabriz. Participants were recruited by a multi-stage sampling method. A total of 1000 participants were included in the study. Data collection was done using a researcher-created questionnaire. Data were analyzed using chi-square test and logistic regression.

**Results: ** The incidence of self-medication was 70.9% for participants who reported illness in the last month. The chance of self-medication was higher in young (P=0.007) and middle-aged (P=0.012) groups, and housewives (P=0.048); and was lower among participants who were not literate (P=0.047). There was no significant relationship between gender and self medication (P=0.553). The high cost of visits was mentioned as a reason for self-medication. The most frequently mentioned drugs used in self-medication were analgesics, cold medicines, and antibiotics, respectively. More frequent reasons for self-medication were the previous experience of the disease, the assumption that the ailment was not important, and the high cost of visits, respectively.

**Conclusion: ** The prevalence of self-medication in this study was high. Considering the results, education in the community, financial support, and monitoring the delivery of drugs can play an important role in improving the pattern of drug use.

## Introduction

Self-medication is an important problematic issue affecting social health worldwide, including Iran.^1^ Several definitions for self-medication are available; according to the World Health Organization (WHO) definition, self-medication is the selection and use of medicines by individuals to treat their self-recognized illnesses or symptoms.^2^

Self-medication may lead to inconsistency between drug prescription patterns and the epidemiological patterns of diseases.^1^Seasonal periodic alterations in the epidemiological status of diseases may alter self-medication patterns.^3^ Laypersons usually carry on self-medication as a constant self-care activity probably due to their inadequate information about drugs side effects.^4^

The operation of medication supply system is a determinative factor that may intervene with the preservation and promotion of the community health.^5^At least one drug may be seen in approximately 75% of therapeutic processes.^1^ Regardless of the enhancement level of the community knowledge about medication, self-medication cannot be completely eliminated.^6^ Lack of acceptance for instructions in prescribing and taking medications in the community have led to a large part of the burden of diseases in Iran (65%).^7^ The differences between drug distribution systems in different countries may impact the prevalence and patterns of self-medication.^1,8,9^ Unfortunately, attempts for regulating drug use have been failed in many cases.^10^ Furthermore, in most health problems, treating the patients is done solely by themselves; according to some estimates, only one twenty-seventh of diseases symptoms are managed by physicians.^11^ The performance of public education programs about the serious consequences of self-medication can be effective in reducing its adverse effects.^12^

Self-medication is a part of self-care. Self-care is defined as what people do in order to stabilize their health.^2^ Different forms of self-medication that have been reported in the literature include use of not-prescribed medications, use of medications prescribed for friends and relatives, medication refills, leftover medications from previous prescriptions, and changing the dosage of prescribed medications.^1,2,9^

Numerous problems that have been related to self-medication include increased risk of adverse drug reactions, drug interactions, drug resistance, disruption in drug distribution systems, increased per capita use of medication in the community,^1^ as well as delay in treatment of serious illnesses, disguising symptoms of acute illnesses and drug interactions and even sudden death in certain cases.^1,3,4,9^ These eventualities are attributable to the prescription of drugs used as an individual’s own initiative and also to the misuse or abuse of over-the-counter (OTC) medicines.^9^

The incidence of self-medication possesses a wide range in developing countries.^10^In Iran, the drug providing systems encounter with excessive misplaced and arbitrary drug use.^8^ Meanwhile, the annual incremental rate of drug consumption in Iran is higher than the average normal growth rate in the world.^10^ Also antibiotic consumption in Iran is higher than other countries.^13,14^ Iran is among the top 20 countries in terms of drug use, and, in Asia, Iran is ranked second, following only China.^15^ The reported prevalence rates of self-medication in Iran settle in a wide range of 12% to 90%, with an average about 53%.^1,7,10,16-18^ Some studies have shown a relatively higher prevalence rate of self-medication among the Iranian community in comparison with other countries.^18^ The prevalence of self-medication in Eastern Mediterranean countries is high, as mentioned above almost in the half of the population in Iran, 42.5% in Jordan, 35.4% in Saudi Arabia, and 68.1% in Pakistan.^19^ According to a systematic review in developing countries, the overall prevalence of self-medication ranged from 8.3% to 87%.^20^ The prevalence of self-medication is not similar in the world so that it has been reported 12.7% in Spain (as a European country), 75% in Chile and 61% in Mexico (as south and central American countries), and 40%-60% in the Vietnamese, 32% in the Chinese, and 71% in the Indians (as Asian countries).^14^ Even in developed countries such as the United States, self-medication is a growing problem due in part to the increasing use of prescription and OTC medications as well as home remedies.^4,10^

The multiple causes of self-medication that have been addressed in various studies include the high cost of treatment, insufficient insurance coverage, having previous experiences with the disease, avoiding the loss of work-time, distrust in health personnel, underrating diseases, and long waiting times for seeing a physician.^1,9,18^ The multiple large-scale surveys undertaken in high-income countries demonstrated a relationship of increased use of OTCs with higher income and education levels.^21^ In many studies, gender, marital status, and the degree of education have been cited as self-medication predictors.^10,17,18^ A long list of self-medication related items reflects the broadness of its determinants range that includes gender, age group, job, marriage status, the degree of education, household income, the degree of individual interest in personal health, demand degree for direct participation in health care decisions, the issues of transportation, health insurance status, and cultural and socio-economic issues.^2,3,5,6,18^

Self-medication is a social phenomenon; therefore, recognizing the determinants and the socio-economic factors of per capita use of drugs, and providing a strategy for reducing and controlling it has a particular importance in pharmaceutical policies.^22^

Moreover, self-medication is considered as an individual behavior configured in the social setting as a relieving agent in confronting with an illness.^9^ People do not exhibit necessarily a similar behavior when encountering an illness. The chosen behavior depends on numerous factors affecting individual behaviors including socioeconomic and cultural factors, the patient’s perceived susceptibility and seriousness of the disease.^21^ The patients’ behavior in making decisions about their health issues is currently changing from passive status to playing an active role in controlling their health.^23^ Different behavioral models have been used as a framework to explain the role of the emerged factors in the occurrence of self-medication behavior. For example, the theory of planned behavior (TPB) has been used extensively for this purpose. According to the TPB, the behavior is linked to an individual’s intention and ability, which are discussed as behavioral intention (BI) and perceived behavioral control (PBC) in this model. As a behavior, contributing factors in self-medication, include the families of the self-medicating people, caregivers, social circle, the environment where they live, and the health system from which they receive services.^9^

The types of sources that are used for risk issues can also influence people’s risk perceptions. For uncertain health risk issues or for abrupt and unexpected natural or human-made risks such as self-medication, people may rely on the opinions of the scientists or experts. A defined format for presenting risk information is numerical estimates. The numeric estimates present risk information with numbers such as the prevalence or incidence rate of a phenomena.^24^ The numerical estimates are derived from researches such as the current study.

“Quaternary prevention” is a “higher” level of prevention. Quaternary prevention is defined as “the action taken to identify people at risk of over-medicalization, to protect them from new medical invasion, and to suggest them interventions ethically acceptable.” The concept of quaternary prevention makes it easier to “identify the patients at risk of over-medicalization.” Therefore, the monitoring of self-medication and identifying its determinants can be considered as important activities in the field of quaternary prevention.^25^

Recognizing the determinants of self-medication is essential for controlling and monitoring it in the community.^14^ Despite a plenty of studies concerning self-medication, it is important to find the basic determinants of self-medication in a country with a good coverage of primary health care. Iran is a country that possesses a good coverage of primary health care based on the international commitments and cooperation with the WHO. Therefore, the results of such a study can be generalized to the other countries worldwide with a good coverage of primary health care. The purpose of this study was to describe self-medication in the practice, to determine the relationship between a number of socio-demographic factors and self-medication and to expand our knowledge about self-medication practices among the population covered by health centers in Tabriz, Iran.

## Materials and Methods

The current study is a descriptive-analytic cross-sectional study that was carried out in 2017 on the population covered by the health centers of Tabriz, North-West of Iran. This study was supported by the Social Determinants of Health Research Center at the Tabriz University of Medical Sciences.

Considering the prevalence of self-medication in the previous studies related to self-medication in Iran (50%), at a 95% confidence interval (alpha = 0.05; z = 1.96) and a precision of 0.05 (d = 0.05) the sample size was calculated as 385. With an estimated 1.5 times the calculated number, the minimum sample size needed was estimated 578. One thousand participants were recruited in this study. Inclusion criteria included being over 15 years of age and remembering personal drug history; exclusion criteria included dissatisfaction to participate in the study.

Sampling was carried out through a multistage method among the population covered by all the 20 health complexes in Tabriz; these health complexes covered 87 health centers. For the first stage, all the health complexes were selected for the sampling process (a census). In the second stage, a random selection was done among the health centers within each of the health complexes. The selection of the health centers was done according to the covered population size so that 2 to 3 centers were selected in each of the complexes; totally 46 of 87 health centers were selected out. In the third stage, a convenience sampling process was conducted among the participants in selected health centers. In allocating the total sample size in any selected health center, a relevant proportion of active household files at any center was considered.

A researcher-created checklist was used to collect the data. This tool included demographic characteristics, names of some drugs and diseases or complaints, and a list of causes for self-medication based on other researches and the study goals. The checklist was sent to experts in community medicine, internal medicine, and pharmacists to assess the content and face validity. The experts confirmed the validity of the checklist. The process of completing the checklist for each participant took about 20 minutes; this process included the explanation and clarification of the purpose of the study by a trained health worker and then answering the items of the checklist by the participants. Informed consent was obtained before the checklist was completed. SPSS version 24 was used to enter and analyze the data.

**Figure 1:**
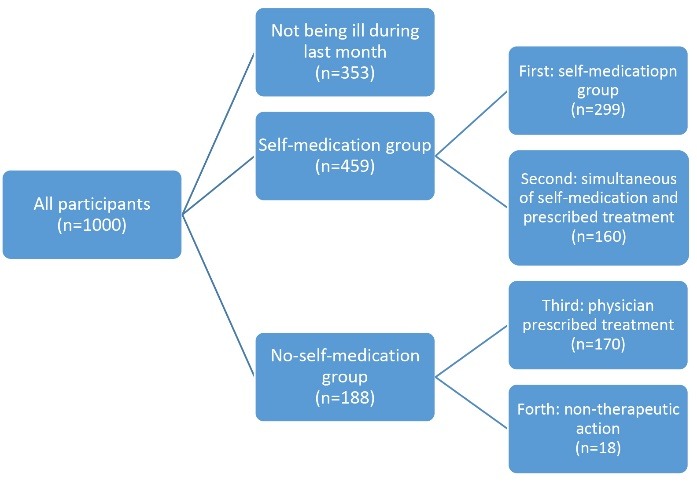
The composition‏ of the participants of the study.

A total of 647 (64.7%) participants (among 1000 recruits) suffered from a disease or illness within a month prior to our visit. We placed these participants in four groups, as follows: the first self-medication group (n=299); the second simultaneous physician’s prescribed treatment and self-medication group (n=160); the third physician’s prescribed treatment group (n=170); and the forth non-therapeutic action group (n=18). By combining the first (self-medication) and the second (simultaneous physician’s prescribed treatment and self-medication) groups, a summation “self-medication” group, was formed, which consisted of 459 participants. 459 participants had either self-medication alone or in combination with a physician’s prescribed treatment. By combining the third (physician’s prescribed treatment) and the fourth (non-therapeutic action) groups, a summation “no-self-medication” group, was formed, which consisted of 188 participants who did not act out self-medication at all. Of the total recruits, 353 had not been ill during the past month, therefore, they were *not* included in either “self-medication” or “no-self-medication” groups. The composition of the participants in the study is shown in Figure 1. All the comparisons were done between “self-medication” group and “no-self-medication” group.

The independent t-test was used to compare the mean age in both groups. The chi-square test and logistic regression were used to determine significant relationships between variables with self-medication and to predict relationships between each of the variables, both individually and after modifying the covariates. In all analyses, the level of statistical significance was considered to be *P*≤0.05.

## Results

All the comparisons performed to achieve results were focused on those participants who were ill during the last month before entering the study and got a treatment (N=647), which included the two groups mentioned above: “self-medication” group (n=459) and “no-self-medication” group (n=188).

*The prevalence of self-medication:* The total number of participants who reported self-medication (as self-medication alone or as simultaneous physician’s prescribed treatment and self-medication) during the past month was 459, representing 45.9% of the total number of recruits and 70.9% of the participants who were ill in the past month. Therefore, the incidence of self-medication based on these findings was 70.9% for participants who suffered from an illness during the last months.

The socio-demographic characteristics of the participants are described in Table 1. Odds ratios and 95% CIs of socio-demographic variables associations with self-medication among the participants are shown in Table 2.

*Age:* The youngest participants were 17 years old (n=3) and the oldest was 78 years old (n=1). The mean age in the self-medication group was 36.45 ± 10.45 years and in the no-self-medication group was 41.95 ± 11.94 years. The mean age of the self-medication group was approximately 5 years less than the no-self-medication group; this difference was statistically significant (*P* < 0.001, t = 4.907; 95% CI: 2.935-6.863). We used the life courses defined in the age classification guideline developed by the Statistical Center of Iran for age grouping the participants in this study; these life courses include childhood, adolescence, youth, middle age, adulthood to the average age of retirement, and retirement (less than one year, 1-14, 15-24, 25-44, 45-64 and 65 years and older, respectively).^26^ In this study, the first two groups were excluded based on the inclusion criteria. The highest proportion of self-medication (75.6%) was found in the “middle age” group. This difference was significant (*P *< 0.001), so that the possibility of self-medication in the “youth” group (15-24) was 5.6 times higher than that seen in the “retired” group (65 years and older), and the possibility of self-medication in the “middle-age” group (25-44) was 3.5 times higher than that seen in the “retired” group (see Table 2).

*Sex:* The prevalence of self-medication was 70.1% in females and 72.3% in males; the difference was not significant. In addition, after adjusting for age, marital status, education level, and type of insurance, the self-medication relationship with gender was not significant (see Table 2).

*Marital status:* Self-medication was seen at similar rates across divorced, widowed, married, and single participants. Almost all the participants were married, so a significant difference was neither expected nor seen (*P *= 0.757).

*Educational levels:* The educational levels of participants, in descending order, include diploma, academic education, primary school, and guidance school, respectively. There was a significant difference in the frequency of self-medication among the different levels of education (*P *= 0.035). By modifying according to the gender, the possibility of self-medication was 2.29 times higher for those with primary school education, 2.42 times higher for those with guidance school educational background, and 2.87 times higher for those with a high school diploma, all in comparison with illiterate people. In analysis using the logistic regression, literacy was seen as a predictor, so that the possibility of self-medication among the participants who were illiterate was about 50% lower than that seen among the group with academic education (see Table 2).

*Job:* In this study, about half of the participants (49.8%) were housewives. The jobs of the participants were categorized into four broad categories: 1. Housewife, 2. Inactive (including unemployed, disabled, and retired), 3. Full-time employees (including health workers, scientific/cultural/artistic, administrative, and military); and 4. Self-employed (including market/service, and hand skills/skilled trades). The highest proportion of self-medication was reported among self-employed group (74.5%) and housewives (72.1%). However, the differences were not significant (*P* = 0.175). By modifying according to the gender variable, the relationship between job and self-medication was significant (*P* = 0.048). The reason for selecting gender as a modifier was the high prevalence of the housewives, who were all females. Thus, the possibility of self-medication among the inactive group and the full-time employees group was lower than that the housewives; the possibility of self-medication among housewives was 2.67 times higher than that seen among the inactive group and 2.09 times higher than that seen among full-time employees group (see Table 2).

*Barriers to access health services:* Of the 459 participants reporting self-medication, the high cost of physicians’ visits was mentioned by 122 (26.6%) and a lack of medical insurance was mentioned by 11 (2.4%) as causes of self-medication and barriers to access health services.

*Drugs:* In this study, the most prevalent drugs used for self-medication included analgesics (79.7%), cold medicines (21.8%), antibiotics (20.3%), herbal medicines (10.5%), gastrointestinal drugs (9.6%), and vitamins and supplements (9.2%).

*Causes:* In this study the most prevalent causes mentioned for self-medication included previous experience with the disease (45.1%), assumption that the disease was not important (41.4%), cost of the physician’s visit (28.6%), and availability of the drug (21.8%). The most-often mentioned illness as a reason for self-medication was a headache (58.2%).

## Discussion

The prevalence of self-medication in this study was 70.9%; inherently and according to the results of other studies, it was estimated high. The prevalence range of self-medication in the world^27-40^ and in Iran^1,5,10,17,18,41-45^ is wide and has been reported from 10% to more than 90%. In a systematic review in Iran, the overall prevalence of self-medication was 53%.^18^ Obviously, the prevalence obtained in this study is much higher.

As mentioned above, an identical prevalence of self-medication cannot be considered in different societies. An expanded spectrum of the percentage of self-medication is plausible because the range of the determinants of self-medication is very wide. The difference between the prevalence of self-medication in various countries may be due to diversity of policies in health systems particularly in regulations, as well as subsidies paid by governments, cultural conditions in the society and, of course, different economic conditions of the communities. Regarding the sample size of the present study and the target population, as well as in comparison to other studies, the obtained prevalence for self-medication in this study seems to be acceptable.

In this study, the mean age of “self-medication” group was about 5 years lower than the “no-self-medication” group; and the possibility of self-medication was higher in the youth and middle-aged groups than in the retired ones. The highest self-medication rate was reported in a study in the age group of 40-60 years^5^ and in another in the over-30 age group.^46^ There were no significant relationships between age and self-medication in the other three studies.^6,10,47^ In general, it can be concluded that there is a great similarity between the results of our study and the results of other studies in terms of the age prevalence of self-medication.

Despite a higher prevalence of self-medication among males in this study, the difference was not significant. After modifying according to the sex variable, there was no significant difference in self-medication rate. In numerous studies, the incidences of self-medication were higher among women.^17,28,48-51^ In a study in Portugal, self-medication was higher among men.^52^ In another study, no significant relationship was found between sex and self-medication.^6^ In general, as mentioned above, some studies showed higher prevalence of self-medication among females, and few others showed reverse results; but in the current study, despite the observation of some differences, no significant difference was obtained. Men and women referring to the health centers may have similar attitudes toward health issues, and therefore gender differences in their treatment approach may not be observed. This may have been due to the development of public health education, which has led to the similarity of men’s and women’ views about health.

There was no significant relationship between self-medication and marriage in this study. In some studies, self-medication was more prevalent in married persons^46^ and in others, it was more prevalent among singles.^17^ In the current study, married people made up about 95% of participants (see Table 1). Therefore, in spite of differences among the frequencies of self-medication by marital status in other studies, in this study, such a difference was not statistically significant. Because the majority of patients in this study were married, significant difference based on marital status for self-medication was not expected; also we did not find it.

In this study, the prevalence of self-medication was higher among highly educated people. The results of some studies show that the highest prevalence of self-medication was among people with academic education (89%).^5^ Our study, as well as some other studies, suggests that people who are less educated may be less confident about self-medication and may feel more need to visit a physician. Meanwhile, the higher education level, the lower need for physician’s visit may result in a higher rate of self-medication.

According to the results of this study, being a housewife (after modifying according to the gender variable) showed a significant relationship with self-medication, so that the possibility of self-medication among housewives was higher than seen among the inactive and full time employees groups. In this study, other jobs did not have a significant relationship with self-medication. In some studies, there has been reported such a relationship^46^ and in some others there has been not reported a significant relationship. It seems that the housewives may have more access to drugs available at home, so the self-medication may be more frequent among them. It seems that the housewives share their experiences about self-medication and somehow they gain a self-confidence and independence of physicians based on their general communications; this process leads to self-medication among them.

One of the most common causes of self-medication in current study was high cost of physicians’ visit in Iran. There are many health insurance organizations in Iran; the Iran Health Insurance Organization has announced that it has provided health insurance for all Iranians. Therefore, the lack of health insurance is mentioned in very few cases as a barrier to access to health services. Here is a contradiction; the insurance for all is provided, on the other hand, the high cost of the visit was reported as a common cause for self-medication. The possible causes of this contradiction include unwillingness to be insured, the insufficient coverage of the costs by health insurance companies, and that people consider the health cost as an extra expending.

In this study, the six most frequently used drugs for self-medication were analgesics, cold medicines, antibiotics, herbal medicines, gastrointestinal drugs, and vitamins and supplements. Analgesics were used much more frequently than the other drugs nearly 80% of self-medication cases. In other studies, analgesics have often been reported as among the most commonly used self-medication drugs.^18^ There is a great similarity between the results of this study and other studies.^53,54^ In most studies in Iran, analgesics are reported as the most commonly used form of self-medication.^5,9,42,46,55^ Many cases of antibiotics use as self-medication have also been reported.^44,46,55^ Generally, it is expected that consumption of analgesics would be high; based on the availability and the presence of most analgesics as the OTC drugs, this issue is justifiable. The more important issue is the relatively high rate of the consumption of antibiotics among the participants (approximately 20%) due to the possibility of increasing antibiotic resistance. The great similarity among the results of the studies implies to some common features of the drugs used for self-medication; the features include availability, lower cost, general acceptability and ability to mitigate an annoying symptom such as pain or fever.

In this study, the most frequently mentioned causes of self-medication included previous experience with the disease, underrating diseases, the high cost of physicians’ visits, and the availability of the drug, respectively. In a systematic review, the most frequently reported cause of self-medication in Iran was feeling mild the symptoms of the disease.^18^ In general, the main causes of self-medication reported in the studies in Iran include the high cost of physicians’ visits, transportation problems, insurance problems, accessibility of medications, cultural and socio-economic issues,^18^ underrating diseases,^5^ previous use of medication that mitigated symptoms, feeling no need for a physician’s visit, assurance about the safety of the drug, lack of health insurance, ease of purchasing medicine from a pharmacy,^10^ availability of drugs,^1^ timing problems,^46^ and prescription of a similar drug by a physician at different visits.^55^ Similarly, the main causes of self-medication reported in the studies worldwide include the impact of information provided in the advertising of antibiotics and antimalarial drugs, the lack of access to health services, inadequate facilities in local health centers, suffering from sexually transmitted diseases,^37^ patients’ inadequate knowledge about their health status and their medication, inadequate adherence to treatment, patient dissatisfaction with the health care provider,^54^ previous prescriptions for a similar illness, underrating the diseases,^27^ high cost of physicians visits, timing problems, and experience with the previous use of drugs. Perhaps this part of the study contains the most important results, since any approach to remediate self-medication should consider these basic issues. As can be seen, there are many similarities in the causes mentioned in the current study, the studies of Iran and the worldwide studies. Therefore, the results of this study can be applied to the proper management of self-medication worldwide. In addition, coherent global management in this regard can result in the synergy of its consequences.

## Conclusion

The high prevalence of self-medication indicates the need to emphasize the importance of the issue and to conduct more specialized studies in health economics, social education, and drug distribution systems related to self-medication. The designing of effective protective systems in order to reduce the out-of-pocket payment for health costs will reduce the cost-related causes of self-medication. It is necessary to increase general health literacy and the community’s awareness about the side effects of drugs and the identification of OTC and non-OTC drugs. In self-medication adjustment approaches, attention should be paid to the various aspects of the issue, including people’s attitudes, the costs of treatment, and drug distribution systems and consequently the availability of drugs.

Considering the above results, the education of the correct use of drugs in the community, financial support and monitoring the delivery and distribution of drugs can play an important role in improving the pattern of drug use.

## Limitations of the study

The design of this study was based on the specified aims of the study. Considering the multidimensional nature of self-medication, further researches could be done with targeted coverage of other dimensions of the subject. In addition, since the sample was selected among the participants who referred to the health centers in Tabriz, individuals who did not seek care at the health centers were not included in this study.

## Implications for practice

The results obtained about the determinants of self-medication in this study can be used for monitoring the status of self-medication in the population covered by primary health care, for prioritizing audiences in general education programs in order to increase the general population health literacy, for comparing the determinants of self-medication phenomenon among the cities of Iran and for designing future studies in order to explore more specific ethnic and cultural determinants. The results of the current study can be used in enhancing public risk perception and through increasing public participation, can promote general education programs in this field. Furthermore, the numerical results obtained will encourage health managers to pay particular attention to self-medication and conducting evidence-based measures in this regard such as the development of the family medicine network. The results of this study are applicable to the enhancement of “quaternary prevention” (the avoidance of over-medicalization) at the community level. This study was conducted in Iran as a country with good coverage of primary health care activities based on the principles of the WHO. Therefore, the results of such a study can be generalized to and utilized by other countries worldwide that have good coverage of primary health care.

## Suggestions

### Policymaking suggestions

The training and management interventions could be facilitated by using the results of the study. Reducing access to medicines through continuous monitoring of drug distribution in pharmacies, raising awareness of physicians, pharmacists and the general public about the rational use of medications, and improving the quality of domestic medications, all can be used to improve the use of medications in Iran. The institutionalization of the family medicine system may also be effective in reducing the burden of self-medication.

### Research suggestions

The performance of future studies could develop a model for including the people who do not refer to health centers; for example, because in spite of the fact that the lack of financial support was mentioned as a barrier to access to health services by those who referred to a health care center in this study, the lack of financial support may be a significant barrier to access to health services for those who do not seek care at the health care centers, too. Finally, with the aim of developing an appropriate model based on the social conditions, it is suggested that the issue of self-medication be studied in a qualitative mixed method study covering the related specialties such as community medicine, psychiatry, psychology, and sociology.

## Ethical approval

This study was approved by the Vice-Chancellor of Ethics of Tabriz University of Medical Sciences under the number IR.TBZMED.REC.1396.163. All participants filled out the informed consent form before completing the checklist.

## Competing interests

The authors declare that they have no competing interests.

## Funding

This study was funded by the Social Determinants of Health Research Center at the Tabriz University of Medical Sciences.

## Authors’ contributions

MA conceptualized and designed the study and guided its approval process. MA encouraged HRSH to investigate demographic determinants of self-medication in the population covered by health centers in Tabriz. MA and HRSH carried out the study. HRSH had primary responsibility for management of data collection. HRSH researched the literature, wrote the literature review, analyzed the data primarily and wrote the manuscript under direct supervision of MA with support from MAJ in data analysis and interpretation. MAJ verified the analytical methods. MA and HRSH finalized the manuscript. All authors discussed and contributed to the final manuscript.

## Acknowledgements

The authors would greatly appreciate all the people who participated in the study for their support and dedication.

**Table 1:** Socio-demographic variables data and their associations with self-medication among the participants

**Variables**			**All participants (Medication group)**				**Participants with a disease in the last month**			
			**Self-medication**	**No-self-medication**	**Without disease**	**Total**	**Self-medication**	**No-self-medication**	**Total**	**P value**
Age group (y)	Youth (15-24)	No.	35	7	50	92	35	7	42	<0.001
		% in each age group	38.0%	7.6%	54.3%	100%	83.3%	16.7%	100%	
		% in each medication group	7.6%	3.7%	14.2%	9.2%	7.6%	3.7%	6.5%	
	Middle age (25-44)	No.	340	110	229	679	340	110	450	
		% in each age group	50.1%	16.2%	33.7%	100%	75.6%	24.4%	100%	
		% in each medication group	74.1%	58.5%	64.9%	67.9%	74.1%	58.5%	69.6%	
	Adulthood (45-64)	No.	76	62	70	208	76	62	138	
		% in each age group	36.5%	29.8%	33.7%	100%	55.1%	44.9%	100%	
		% in each medication group	16.6%	33.0%	19.8%	20.8%	16.6%	33.0%	21.3%	
	Retired (≥65)	No.	8	9	4	21	8	9	17	
		% in each age group	38.1%	42.9%	19.0%	100%	47.1%	52.9%	100%	
		% in each medication group	1.7%	4.8%	1.1%	2.1%	1.7%	4.8%	2.6%	
Sex	Female	No.	284	121	175	580	284	121	405	0.553
		% in each sex group	49.0%	20.9%	30.2%	100%	70.1%	29.9%	100%	
		% in each medication group	61.9%	64.4%	49.6%	58.0%	61.9%	64.4%	62.6%	
	Male	Frequency	175	67	178	420	175	67	242	
		% in each sex group	41.7%	16.0%	42.4%	100%	72.3%	27.7%	100%	
		% in each medication group	38.1%	35.6%	50.4%	42.0%	38.1%	35.6%	37.4%	
Marital status	Single	Frequency	11	3	20	34	11	3	14	0.757
		% in each marital status group	32.4%	8.8%	58.8%	100%	78.6%	21.4%	100%	
		% in each medication group	2.4%	1.6%	5.7%	3.4%	2.4%	1.6%	2.2%	
	Married	Frequency	433	180	331	944	433	180	613	
		% in each marital status group	45.9%	19.1%	35.1%	100%	70.6%	29.4%	100%	
		% in each medication group	94.3%	95.7%	93.8%	94.4%	94.3%	95.7%	94.7%	
	Widowed	Frequency	9	4	2	15	9	4	13	
		% in each marital status group	60.0%	26.7%	13.3%	100%	69.2%	30.8%	100%	
		% in each medication group	2.0%	2.1%	0.6%	1.5%	2%	2.1%	2%	
	Divorced	Frequency	6	1	0	7	6	1	7	
		% in each marital status group	85.7%	14.3%	0.0%	100%	85.7%	14.3%	100%	
		% in medication group	1.3%	0.5%	0.0%	0.7%	1.3%	5%	1.1%	
Education levels	Not literate	Frequency	21	20	6	47	21	20	41	0.035
		% in each education level group	44.7%	42.6%	12.8%	100%	51.2%	48.8%	100%	
		% in each medication group	4.6%	10.6%	1.7%	4.7%	4.6%	10.6%	6.3%	
	Primary school	Frequency	87	36	48	171	87	36	123	
		% in each education level group	50.9%	21.1%	28.1%	100%	70.7%	29.3%	100%	
		% in each medication group	19.0%	19.1%	13.6%	17.1%	19%	19.1%	19%	
	Guidance school	Frequency	83	32	51	166	83	32	115	
		% in each education level group	50.0%	19.3%	30.7%	100%	72.2%	27.8%	100%	
		% in each medication group	18.1%	17.0%	14.4%	16.6%	18.1%	17%	17.8%	
	Diploma	Frequency	179	59	167	405	179	59	238	
		% in each education level group	44.2%	14.6%	41.2%	100%	75.2%	24.8%	100%	
		% in each medication group	39.0%	31.4%	47.3%	40.5%	39%	31.4%	36.8%	
	Academic education	Frequency	89	41	81	211	89	41	130	
		% in each education level group	42.2%	19.4%	38.4%	100%	68.5%	31.5%	100%	
		% in each medication group	19.4%	21.8%	22.9%	21.1%	19.4%	21.8%	20.1%	
Job	Housewives	Frequency	253	98	147	498	253	98	351	0.175
		% in each job group	50.8%	19.7%	29.5%	100%	72.1%	27.9%	100%	
		% in each medication group	55.1%	52.1%	41.6%	49.8%	55.1%	52.1%	54.6%	
	Inactive	Frequency	28	17	18	63	28	17	45	
		% in each job group	44.4%	27.0%	28.6%	100%	62.2%	37.8%	100%	
		% in each medication group	6.1%	9.0%	5.1%	6.3%	6.1%	9%	7%	
	Full-time employees	Frequency	55	31	56	142	55	31	86	
		% in each job group	38.7%	21.8%	39.4%	100%	64%	36%	100%	
		% in each medication group	12.0%	16.5%	15.9%	14.2%	12%	16.5%	13.3%	
	Self-employed	Frequency	123	42	132	297	123	42	165	
		% in each job group	41.4%	14.1%	44.4%	100%	74.5%	25.5%	100%	
		% in each medication group	26.8%	22.3%	37.4%	29.7%	26.8%	22.3%	25.5%	

**Table 2:** Odds Ratios and 95% CIs of socio-demographic variables associations with self-medication among the participants

**Variables**			***P*** ** value**	**Odds Ratio**	**95% CI**	
					**Lower**	**Upper**
Age group (y)		Youth (15-24)	0.007	5.625	1.610	19.657
		Middle age (25-44)	0.012	3.477	1.310	9.231
		Adulthood (45-64)	0.533	1.379	0.502	3.785
		Retired (≥65)	Reference			
Sex	Sex alone	Female	0.533	0.899	0.631	1.279
		Male	Reference			
	Sex together with confounders	Female	0.110	0.727	0.491	1.075
		Male	Reference			
Marital status		Single	0.696	1.636	0.138	19.387
		Married	0.399	2.494	0.298	20.866
		Widowed	0.427	0.427	0.237	30.066
		Divorced	Reference			
Education level	Education level alone	Not literate	0.047	0.484	0.237	0.989
		Primary school	0.695	1.113	0.651	1.904
		Guidance school	0.526	1.195	0.689	2.072
		Diploma	0.165	1.398	0.871	2.242
		Academic education	Reference			
	Education level together with confounders	Not literate	Reference			
		Primary school	0.025	2.293	1.110	4.763
		Guidance school	0.018	2.429	1.161	5.082
		Diploma	0.002	2.871	1.455	5.666
		Academic education	0.055	2.022	0.985	4.153
Job together with confounders		Housewives	Reference			
		Inactive	0.026	0.375	0.158	0.888
		Full-time employees	0.021	0.477	0.254	0.896
		Self-employed	0.247	0.683	0.345	1.352
